# Expert consensus on the diagnosis and treatment of *NTRK* gene fusion solid tumors in China

**DOI:** 10.1111/1759-7714.14644

**Published:** 2022-09-20

**Authors:** Chunwei Xu, Lu Si, Wenxian Wang, Ziming Li, Zhengbo Song, Qian Wang, Aijun Liu, Jinpu Yu, Wenfeng Fang, Wenzhao Zhong, Zhijie Wang, Yongchang Zhang, Jingjing Liu, Shirong Zhang, Xiuyu Cai, Anwen Liu, Wen Li, Ping Zhan, Hongbing Liu, Tangfeng Lv, Liyun Miao, Lingfeng Min, Yu Chen, Jingping Yuan, Feng Wang, Zhansheng Jiang, Gen Lin, Xingxiang Pu, Rongbo Lin, Weifeng Liu, Chuangzhou Rao, Dongqing Lv, Zongyang Yu, Lei Lei, Xiaoyan Li, Chuanhao Tang, Chengzhi Zhou, Junping Zhang, Junli Xue, Hui Guo, Qian Chu, Rui Meng, Jingxun Wu, Rui Zhang, Xiao Hu, Jin Zhou, Zhengfei Zhu, Yongheng Li, Hong Qiu, Fan Xia, Yuanyuan Lu, Xiaofeng Chen, Rui Ge, Enyong Dai, Yu Han, Weiwei Pan, Jiancheng Luo, Hongtao Jia, Xiaowei Dong, Fei Pang, Kai Wang, Liping Wang, Youcai Zhu, Yanru Xie, Xinqin Lin, Jing Cai, Jia Wei, Fen Lan, Huijing Feng, Lin Wang, Yingying Du, Wang Yao, Xuefei Shi, Xiaomin Niu, Dongmei Yuan, Yanwen Yao, Jianhui Huang, Yinbin Zhang, Pingli Sun, Hong Wang, Mingxiang Ye, Dong Wang, Zhaofeng Wang, Bing Wan, Donglai Lv, Qing Wei, Jin Kang, Jiatao Zhang, Chao Zhang, Genhua Yu, Juanjuan Ou, Lin Shi, Zhongwu Li, Zhefeng Liu, Jing Liu, Nong Yang, Lin Wu, Huijuan Wang, Gu Jin, Liu Yang, Guansong Wang, Meiyu Fang, Yong Fang, Yuan Li, Xiaojia Wang, Yiping Zhang, Shenglin Ma, Biyun Wang, Xiaotian Zhang, Yong Song, Yuanzhi Lu

**Affiliations:** ^1^ Institute of Cancer and Basic Medicine (ICBM) Chinese Academy of Sciences Hangzhou People's Republic of China; ^2^ Department of Respiratory Medicine, Affiliated Jinling Hospital Medical School of Nanjing University Nanjing People's Republic of China; ^3^ Key Laboratory of Carcinogenesis and Translational Research (Ministry of Education/Beijing), Department of Melanoma and Sarcoma Peking University Cancer Hospital and Institute Beijing People's Republic of China; ^4^ Department of Chemotherapy Chinese Academy of Sciences University Cancer Hospital (Zhejiang Cancer Hospital) Hangzhou People's Republic of China; ^5^ Department of Shanghai Lung Cancer Center, Shanghai Chest Hospital Shanghai Jiao Tong University Shanghai People's Republic of China; ^6^ Department of Respiratory Medicine Affiliated Hospital of Nanjing University of Chinese Medicine, Jiangsu Province Hospital of Chinese Medicine Nanjing People's Republic of China; ^7^ Senior Department of Pathology The 7th Medical Center of PLA General Hospital Beijing People's Republic of China; ^8^ Cancer Molecular Diagnostics Core Tianjin Medical University Cancer Institute and Hospital Tianjin People's Republic of China; ^9^ Department of Medical Oncology Sun Yat‐sen University Cancer Center, State Key Laboratory of Oncology in South China, Collaborative Innovation Center for Cancer Medicine Guangzhou People's Republic of China; ^10^ Guangdong Lung Cancer Institute, Guangdong Provincial Laboratory of Translational Medicine in Lung Cancer Guangdong Provincial People's Hospital, Guangdong Academy of Medical Sciences, School of Medicine Guangzhou People's Republic of China; ^11^ State Key Laboratory of Molecular Oncology, Department of Medical Oncology, National Cancer Center/National Clinical Research Center for Cancer/Cancer Hospital Chinese Academy of Medical Sciences and Peking Union Medical College Beijing People's Republic of China; ^12^ Department of Medical Oncology, Lung Cancer and Gastrointestinal Unit Hunan Cancer Hospital/The Affiliated Cancer Hospital of Xiangya School of Medicine, Central South University Changsha People's Republic of China; ^13^ Department of Thoracic Cancer Jilin Cancer Hospital Changchun People's Republic of China; ^14^ Translational Medicine Research Center, Key Laboratory of Clinical Cancer Pharmacology and Toxicology Research of Zhejiang Province, Affiliated Hangzhou First People's Hospital, Cancer Center Zhejiang University School of Medicine Hangzhou People's Republic of China; ^15^ Department of VIP Inpatient Sun Yet‐Sen University Cancer Center, State Key Laboratory of Oncology in South China, Collaborative Innovation Center for Cancer Medicine Guangzhou People's Republic of China; ^16^ Department of Oncology Second Affiliated Hospital of Nanchang University Nanchang People's Republic of China; ^17^ Key Laboratory of Respiratory Disease of Zhejiang Province, Department of Respiratory and Critical Care Medicine Second Affiliated Hospital of Zhejiang University School of Medicine, Cancer Center, Zhejiang University Hangzhou People's Republic of China; ^18^ Department of Respiratory Medicine Affiliated Drum Tower Hospital, Medical School of Nanjing University Nanjing People's Republic of China; ^19^ Department of Respiratory Medicine Clinical Medical School of Yangzhou University, Subei People's Hospital of Jiangsu Province Yangzhou People's Republic of China; ^20^ Department of Medical Oncology Fujian Medical University Cancer Hospital and Fujian Cancer Hospital Fuzhou People's Republic of China; ^21^ Department of Pathology Renmin Hospital of Wuhan University Wuhan People's Republic of China; ^22^ Department of Internal Medicine, Cancer Center of PLA, Qinhuai Medical Area Affiliated Jinling Hospital, Medical School of Nanjing University Nanjing People's Republic of China; ^23^ Department of Integrative Oncology Tianjin Medical University Cancer Institute and Hospital Tianjin People's Republic of China; ^24^ Department of Medical Oncology, Lung Cancer and Hunan Cancer Hospital/The Affiliated Cancer Hospital of Xiangya School of Medicine Central South University Changsha People's Republic of China; ^25^ Department of Orthopaedic Oncology Surgery, Beijing Ji Shui Tan Hospital Peking University Beijing People's Republic of China; ^26^ Department of Radiotherapy and Chemotherapy, Hwamei Hospital University of Chinese Academy of Sciences Ningbo People's Republic of China; ^27^ Department of Pulmonary Medicine Taizhou Hospital of Wenzhou Medical University Taizhou People's Republic of China; ^28^ Department of Respiratory Medicine, The 900th Hospital of the Joint Logistics Team (The Former Fuzhou General Hospital) Fujian Medical University Fuzhou People's Republic of China; ^29^ Department of Oncology, Beijing Tiantan Hospital Capital Medical University Beijing People's Republic of China; ^30^ Department of Medical Oncology Peking University International Hospital Beijing People's Republic of China; ^31^ State Key Laboratory of Respiratory Disease, National Clinical Research Center for Respiratory Disease, Guangzhou Institute of Respiratory Health The First Affiliated Hospital of Guangzhou Medical University (The First Affiliated Hospital of Guangzhou Medical University) Guangzhou People's Republic of China; ^32^ Department of Thoracic Oncology Shanxi Academy of Medical Sciences, Shanxi Bethune Hospital Taiyuan People's Republic of China; ^33^ Department of Oncology, Shanghai East Hospital, School of Medicine Tongji University Shanghai People's Republic of China; ^34^ Department of Medical Oncology The First Affiliated Hospital of Xi'an Jiaotong University Xi'an People's Republic of China; ^35^ Department of Oncology, Tongji Hospital of Tongji Medical College Huazhong University of Science and Technology Wuhan People's Republic of China; ^36^ Cancer Center, Union Hospital, Tongji Medical College Huazhong University of Science and Technology Wuhan People's Republic of China; ^37^ Department of Medical Oncology, The First Affiliated Hospital of Medicine Xiamen University Xiamen People's Republic of China; ^38^ Department of Medical Oncology Cancer Hospital of China Medical University Shenyang People's Republic of China; ^39^ Zhejiang Key Laboratory of Radiation Oncology Cancer Hospital of the University of Chinese Academy of Sciences (Zhejiang Cancer Hospital) Hangzhou People's Republic of China; ^40^ Department of Medical Oncology, Sichuan Cancer Hospital and Institute, Sichuan Cancer Center, School of Medicine University of Electronic Science and Technology Chengdu People's Republic of China; ^41^ Department of Radiation Oncology Fudan University Shanghai Cancer Center Shanghai People's Republic of China; ^42^ Key Laboratory of Carcinogenesis and Translational Research (Ministry of Education/Beijing), Department of Radiation Oncology Peking University Cancer Hospital and Institute Beijing People's Republic of China; ^43^ State Key Laboratory of Cancer Biology, National Clinical Research Center for Digestive Diseases and Xijing Hospital of Digestive Diseases Fourth Military Medical University Xi'an People's Republic of China; ^44^ Department of Oncology Jiangsu Province Hospital and Nanjing Medical University First Affiliated Hospital Nanjing People's Republic of China; ^45^ Department of General Surgery Huadong Hospital Affiliated to Fudan University Shanghai People's Republic of China; ^46^ Department of Oncology and Hematology China‐Japan Union Hospital of Jilin University Changchun People's Republic of China; ^47^ Department of Gastrointestinal Oncology Harbin Medical University Cancer Hospital Harbin People's Republic of China; ^48^ Department of Cell Biology, College of Medicine Jiaxing University Jiaxing People's Republic of China; ^49^ Aiyi Technology Co., Ltd Beijing People's Republic of China; ^50^ Department of Pathology Shanghai OrigiMed Co, Ltd Shanghai People's Republic of China; ^51^ Department of Oncology Baotou Cancer Hospital Baotou People's Republic of China; ^52^ Department of Thoracic Disease Diagnosis and Treatment Center, Zhejiang Rongjun Hospital The Third Affiliated Hospital of Jiaxing University Jiaxing People's Republic of China; ^53^ Department of Oncology Lishui Municipal Central Hospital Lishui People's Republic of China; ^54^ Department of the Comprehensive Cancer Center Affiliated Drum Tower Hospital, Medical School of Nanjing University Nanjing People's Republic of China; ^55^ Department of Pathology Shanxi Academy of Medical Sciences, Shanxi Bethune Hospital Taiyuan People's Republic of China; ^56^ Department of Oncology The First Affiliated Hospital of Anhui Medical University Hefei People's Republic of China; ^57^ Department of Interventional Oncology The First Affiliated Hospital, Sun Yat‐sen University Guangzhou People's Republic of China; ^58^ Department of Respiratory Medicine, Huzhou Hospital Zhejiang University School of Medicine Huzhou People's Republic of China; ^59^ Department of Oncology, The Second Affiliated Hospital of Medical College Xi'an Jiaotong University Xi'an People's Republic of China; ^60^ Department of Pathology The Second Hospital of Jilin University Changchun People's Republic of China; ^61^ Senior Department of Oncology The 5th Medical Center of PLA General Hospital Beijing People's Republic of China; ^62^ Department of Respiratory Medicine The Affiliated Jiangning Hospital of Nanjing Medical University Nanjing People's Republic of China; ^63^ Department of Clinical Oncology The 901 Hospital of Joint Logistics Support Force of People Liberation Army Hefei People's Republic of China; ^64^ Department of Radiation Oncology Zhebei Mingzhou Hospital Huzhou People's Republic of China; ^65^ Department of Oncology and Southwest Cancer Center, Southwest Hospital Third Military Medical University (Army Medical University) Chongqing People's Republic of China; ^66^ Department of Respiratory Medicine Zhongshan Hospital, Fudan University Shanghai People's Republic of China; ^67^ Key Laboratory of Carcinogenesis and Translational Research (Ministry of Education/Beijing), Department of Pathology Peking University Cancer Hospital and Institute Beijing People's Republic of China; ^68^ Department of Oncology, Ruijin Hospital Shanghai Jiao tong University School of Medicine Shanghai People's Republic of China; ^69^ Department of Internal Medicine The Affiliated Cancer Hospital of Zhengzhou University, Henan Cancer Hospital Zhengzhou People's Republic of China; ^70^ Department of Bone and Soft‐Tissue Surgery Chinese Academy of Sciences University Cancer Hospital (Zhejiang Cancer Hospital) Hangzhou People's Republic of China; ^71^ Key Laboratory of Tumor Molecular Diagnosis and Individualized Medicine of Zhejiang Province Zhejiang Provincial People's Hospital, People's Hospital of Hangzhou Medical College Hangzhou People's Republic of China; ^72^ Institute of Respiratory Diseases, Xinqiao Hospital Third Military Medical University Chongqing People's Republic of China; ^73^ Department of Medical Oncology, Sir Run Run Shaw Hospital Zhejiang University Hangzhou People's Republic of China; ^74^ Department of Pathology Fudan University Shanghai Cancer Center Shanghai People's Republic of China; ^75^ Department of Oncology, Key Laboratory of Clinical Cancer Pharmacology and Toxicology, Research of Zhejiang Province Affiliated Hangzhou Cancer Hospital, Cancer Center, Zhejiang University School of Medicine Hangzhou People's Republic of China; ^76^ Department of Breast Cancer and Urological Medical Oncology Fudan University Shanghai Cancer Center, Department of Oncology, Shanghai Medical College, Fudan Unviersity Shanghai People's Republic of China; ^77^ Key Laboratory of Carcinogenesis and Translational Research (Ministry of Education/Beijing), Department of Gastrointestinal Oncology Peking University Cancer Hospital and Institute Beijing People's Republic of China; ^78^ Department of Clinical Pathology The First Affiliated Hospital of Jinan University Guangzhou People's Republic of China

**Keywords:** fusion, precision medicine, solid tumor, targeted therapy, tyrosine receptor kinase

## Abstract

Gene fusions can drive tumor development for multiple types of cancer. Currently, many drugs targeting gene fusions are being approved for clinical application. At present, tyrosine receptor kinase (TRK) inhibitors targeting *neurotrophic tyrosine receptor kinase* (*NTRK*) gene fusions are among the first “tumor agnostic” drugs approved for pan‐cancer use. Representative TRK inhibitors, including larotrectinib and entrectinib, have shown high efficacy for many types of cancer. At the same time, several second‐generation drugs designed to overcome first‐generation drug resistance are undergoing clinical development. Due to the rarity of *NTRK* gene fusions in common cancer types and technical issues regarding the complexity of fusion patterns, effectively screening patients for TRK inhibitor treatment in routine clinical practice is challenging. Different detection methods including immunohistochemistry, fluorescence in situ hybridization, reverse transcription‐polymerase chain reaction, and (DNA and/or RNA‐based) next‐generation sequencing have pros and cons. As such, recommending suitable tests for individual patients and ensuring the quality of tests is essential. Moreover, at present, there is a lack of systematic review for the clinical efficacy and development status of first‐ and second‐generation TRK inhibitors. To resolve the above issues, our expert group has reached a consensus regarding the diagnosis and treatment of *NTRK* gene fusion solid tumors, aiming to standardize clinical practice with the goal of benefiting patients with *NTRK* gene fusions treated with TRK inhibitors.

## Graphical abstract

To improve the diagnosis and treatment of *NTRK* gene fusion solid tumors in China, we reached consensus on detection strategy based on cancer type classification, quality control requirements, treatment and clinical management. Moreover, we systematically reviewed the clinical efficacy, resistance mechanisms and development status of TRK inhibitors.

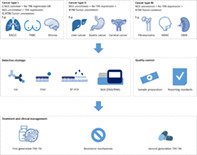

## INTRODUCTION

Tyrosine kinase receptor (TRK) inhibitors targeting *neurotrophic tyrosine receptor kinase* (*NTRK*) gene fusions are the first “tumor agnostic” drugs approved for pan‐cancer use. To standardize the screening of *NTRK* gene fusions and the clinical application of TRK inhibitors, we established a generalized, systematic strategy for the diagnosis and treatment of *NTRK* gene fusion in solid tumor patients. There are three key features of our recommendations: (1) To reasonable assign appropriate test to patients with different cancer types, we classify cancer types into three types based on: (i) whether next‐generation sequencing (NGS) is commonly performed, (ii) whether TRK protein is physiologically expressed, and (iii) whether *NTRK* gene fusion is prevalent. Specific detection strategy is suggested according to this classification. (2) To ensure the detection quality, the sample requirements, the laboratory standards, and the reporting criteria are listed and reconfirmation test is suggested in appropriate scenarios. (3) To guide treatment decision, a DNA‐based NGS test is suggested when disease progress after TRK inhibitor treatment, and the clinical efficacy of major TRK inhibitors, the drug resistance mechanisms, and the first‐ and second‐ generation TRK inhibitors under development are summarized.

## THE BIOLOGICAL BASIS OF THE *NTRK* GENE

### The gene structures and biological functions of the *NTRK* gene

**FIGURE 1:**
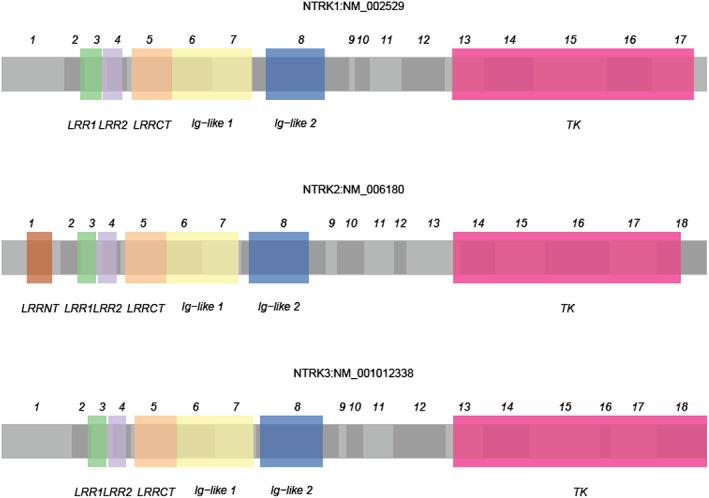
The structure of *NTRK1/2/3* gene coding region. LRR1, leucine‐rich repeat 1 domain; LRR2, leucine‐rich repeat 2 domain; LRRCT, leucine‐rich repeat C‐terminal domain; LRRNT, leucine‐rich repeat N‐terminal domain; TK, tyrosine kinase domain. To to clearly show different parts of the structure, LRRNT, LRR1, LRR2, LRRCT, Ig‐like 1, Ig‐like 2 and TK

*NTRK 1/2/3* genes, respectively, encode the tyrosine kinase receptor (TRK) A/B/C. These genes are located on human chromosomes 1q23.1, 9q21.33, and 15q25.3.^1^ TRK receptor proteins are commonly expressed in human nerve tissues, smooth muscles, and the testes. These proteins can be activated by neurotrophins such as NT‐3/4 etc., leading to self‐dimerization and phosphorylation, and subsequently activating several downstream signaling pathways such as the MAPK pathway, the PI3K‐AKT pathway, and the PLCγ pathway.^1^ TRK receptor proteins play an important role in normal neuron functioning, including in neuronal cell differentiation and proliferation, and in the survival and formation of axons, dendrites, and synapses. TRK receptor proteins also participate in neurophysiological functioning for pain, proprioception, appetite, and memory regulations; and participate in various types of neuron protection such as ischemia.^2^, ^3^ Although the three *NTRK* genes are located at different positions on different chromosomes, they are highly homologous and nearly have the same structure (Figure 1).^4^

### *NTRK* gene fusion and its mechanism as a driver mutation

Gene fusions are a common type of driver mutation in solid tumors.^5^ In many types of adult and pediatric malignancies, *NTRK*‐related intra‐ or interchromosomal structural variants are key oncogenic factors. The most common scenario is chromosomal structural variants at the DNA level that lead to the formation of gene fusion at the downstream RNA level. In general, the partner gene serves as the 5′‐terminal gene and provides the promoter region (or enhancer region), and the *NTRK1/2/3* gene serves as the 3′‐terminal gene and provides the kinase domain. The breakpoint of the *NTRK* gene fusion usually does not involve frameshift mutation (in‐frame). Creating a complete *NTRK* kinase domain without frameshift variants is the critical part of oncogenicity for *NTRK* gene fusions.^6^ Oncogenic *NTRK* gene fusions cause the overexpression and sustained activation of TRK kinase, inducing sustained activation of TRK receptors, independent receptor dimerization caused by ligand‐binding or other mechanisms. The activation of TRK receptors then leads to excessive cell proliferation and apoptotic resistance via the sustained activation of downstream signaling pathways such as MAPK/MEK/ERK and PI3K/AKT/mTOR, promoting tumorigenesis and development.^1^, ^7^

### Prevalence of *NTRK* gene fusions and their partner genes in different cancer types

Past research indicated that *NTRK* gene fusions are present in at least 45 cancer types, with an overall mutational frequency of 0.3% for multiple partner genes.^8^ A meta‐analysis had reported the mutational frequency of *NTRK* gene fusions in different cancer types,^9^ of which, that in secretory breast cancer, secretory salivary gland carcinoma, and infantile fibrosarcoma achieved >75%, but that in common cancer types was low (Table 1). The Asian population, especially in eastern and southern Asian, has a relatively higher mutational frequency of *NTRK* gene fusions (0.4%) as compared to other races.^8^ The numbers of partner genes for *NTRK1/2/3* genes reported to date are at least 94, 39, and 61, respectively (Supporting Information Table S1).

**TABLE 1:** The mutational frequency of *NTRK* gene fusions in pan‐cancer tissues^10^

Mutational frequency of *NTRK* gene fusions	Adult/pediatric	Cancer type
<5%	Adult	Colorectal cancer, glioma, melanoma, lung cancer, pancreatic cancer, cholangiocarcinoma, various types of sarcomas
	Pediatric	Glioma, various types of sarcomas
5%–75%	Adult	Thyroid adenocarcinoma, gastrointestinal stromal tumor
	Pediatric	Thyroid adenocarcinoma, congenital mesoblastic nephroma, spitzoid melanoma
>75%	Adult	Secretory salivary gland carcinoma, secretory breast cancer
	Pediatric	Infantile fibrosarcoma, secretory breast cancer

*NTRK* gene fusions are prevalent in *BRAF* wild‐type, *MLH1* promoter hypermethylated non‐Lynch syndrome, microsatellite instability‐high colorectal cancer patients.^11^ It is noteworthy that the fusions of the three *NTRK* genes are mutually exclusive. In cancers including adult osteosarcoma, breast cancer, colorectal cancer, etc., *NTRK* gene fusions are generally mutually exclusive for major driver mutations such as *KRAS*, *APC*, *TP53*, *PIK3CA*, *BRAF*, and *SMAD4*.^8^

### Somatic and other *NTRK* gene variants

Currently, only fusion variants are “actionable” for *NTRK* genes.^12^ However, the oncogenicity of other *NTRK* variant types, including somatic mutations, splice variants, and amplifications, is also being studied. In past research, *NTRK* substitutions and insertion/deletion mutations have been determined in ovarian cancer, colorectal cancer, lung cancer, melanoma, and myeloid lymphoma;^13^, ^14^, ^15^, ^16^, ^17^ however, it is still unclear whether or not the majority of these mutations lead to tumorigenesis and cancer development. Overall, most *NTRK* substitution mutations are yet not druggable, although *NTRK1* G667C and *NTRK3* G696A have been proven to cause a resistance to TRK inhibitors.^18^ Other *NTRK* variant types, such as the *NTRK1* in‐frame deletion (ΔTRKA) in acute myeloid lymphoma, the *NTRK1* splice variant (TRKAIII) in neuroblastoma, TRKA overexpression in breast cancer, TRKB overexpression in neuroblastoma, and TRKC overexpression in small round cell tumor may possibly be related to tumorigenesis and cancer development.^19^, ^20^, ^21^

## TYPES OF DETECTION METHODS AND THEIR LIMITATIONS

### Immunohistochemistry (IHC)

Immunohistochemistry is fast, low‐cost, and convenient, and requires relatively fewer tumor tissues, making it suitable for large‐scale screening in cancer types with a low mutational frequency of *NTRK* gene fusions. The FDA, CE IVD, and NMPA approved antibody EPR17341, which can bind to a common antigen at the C‐terminal domain of all three types of TRK proteins, has been widely applied in clinical practice. Pan‐TRK IHC possesses a relatively high sensitivity and specificity. Different *NTRK* genes, in particular, display contrasting sensitivity. The sensitivity of *NTRK3* (55%–79%) is lower compared to *NTRK1* (88%–96%) and *NTRK2* (89%–100%).^22^, ^23^ The specificity in different cancer types also varies. The specificity of IHC in lung, intestinal, thymus, pancreas, and biliary tract cancers is 100%, while that for breast cancer and salivary gland carcinoma are 82 and 52%, respectively.^23^

To avoid false‐negatives and a loss for further confirmation testing, test sensitivity is important. The standardization of operating procedures can enhance detection accuracy. Theoretically, most normal cells, with the exception of nerve tissues, smooth muscles, and tissues within the testes, do not express TRK proteins.^24^, ^25^, ^26^, ^27^ When tumor cells harbor *NTRK* gene fusions, TRK protein expression increases. Testes tissues, nerve tissues, and cell lines, including KM12 (*TPM3‐NTRK1*),^28^ MO‐91 (*ETV6‐NTRK3*), and CUTO‐3.29 (*MPRIP‐NTRK1*)^29^ can serve as a positive external control. Peripheral nerve cells present in stroma can serve as an internal control. Nontumor cells such as skin, vessel, and inflammatory cells can serve as a negative internal control.^30^ Different *NTRK* genes and partner genes possess distinct expression patterns. Typically, diffuse cytoplasmic expression with the pan‐TRK antibody EPR17341 indicates *NTRK1/2* gene fusion. In contrast, TRKC expression is generally weaker and is located at the nucleus.^30^ Greater than 1% positive cells can be defined as IHC pan‐TRK positive.

Pan‐TRK IHC is not recommended as the screening method for nerve‐derived tumors or smooth muscle/nerve‐differentiated tumors. Importantly, pan‐TRK IHC can only be used in the screening of *NTRK* gene fusions, but not in confirmations. Following transcription and expression, it is theoretically possible to detect fusion protein. Thus IHC can be used as a reconfirmation method for other test results. For example, in a larotreactinib clinical trial,^31^ six patients experienced a best response for progressive disease (PD). For the remaining five patients, with the exception of one patient with acquired resistance, only three patients were able to provide sufficient tumor tissue specimens for pan‐TRK IHC testing. Results obtained from testing were all negative, indicating the importance of IHC reconfirmation.

### Fluorescence in situ hybridization (FISH)

Fluorescence in situ hybridization is a common method for detecting chromosomal structural variants such as *ALK*, *ROS1*, and *RET* gene fusions. The advantages of FISH include a short turnaround period, a wide application field, and a relatively low cost. However, regards to *NTRK1/2/3*: three genes and over 100 known partner genes, FISH requires at least three tests, which results in a low efficiency and a high price. Amplification of the FISH probe region of *NTRK* genomic location may lead to false‐positive; Intrachromosomal *NTRK* gene fusion with the location of *NTRK* gene nearby that of the partner gene may lead to false‐negative (particularly in *NTRK1* gene fusions where short reversions and intra‐chromosomal rearrangements are common).^32^, ^33^, ^34^ FISH detection of *ETV6‐NTRK3* fusion can be used as a confirmation method for fusion‐prevalent tumors.

The diagnostic criteria of FISH in detecting *NTRK* gene fusions is similar to that for other gene fusions. To avoid false‐positive signals due to human error, the slice thickness should be 4 μM, and counting for at least 50 fluorescent signals in randomly selected, nonoverlapping tumor cell nuclei should be performed by at least one expert. Positive cutoff thresholds can be 10% or 15%.^35^ Here, it should be noted that FISH possesses the following limitations: (1) FISH requires an expert for the interpretation of results, (2) Fish has a high false‐negative rate, and (3) FISH cannot classify in‐frame and out‐frame fusions.

### Reverse transcription‐polymerase chain reaction (RT‐PCR)

Reverse transcription‐polymerase chain reaction can qualitatively and quantitatively measure the RNA transcription products of gene fusions. The technique is relatively cheap and requires a relatively short test period. To perform RT‐PCR, designing upstream and downstream primers according to known fusion positions is required, and thus RT‐PCR is only applicable for detecting known fusions. Unfortunately, there are over 100 known partner genes for *NTRK*, and a significant difference is observed for both breakpoint positions and for the exons of involving genes. The *ETV6‐NTRK3* fusion that frequently occurs in infantile fibrosarcoma, secretory breast cancer, and congenital mesoblastic nephroma can be tested using RT‐PCR.^36^, ^37^, ^38^, ^39^

### Next‐generation sequencing (NGS)

Since the advancement of precision medicine, multiple genomic biomarkers, including *NTRK* gene fusions, have become the key element in guiding cancer treatment. Next‐generation sequencing (NGS) is currently the only technique that can simultaneously detect multiple types of variants, including fusions, mutations, amplifications, etc., and detect tens, hundreds, or even tens of thousands of genes at once, with high sensitivity and specificity. The FDA and NMPA have approved several panels that include *NTRK* gene fusions for clinical application. Considering low mutational frequency in common cancer types, numerous partner genes, and the complex fusion structure of *NTRK* gene fusions, NGS is the most crucial and valuable technique for detecting *NTRK* gene fusions. However, NGS is relatively complicated, and requires a longer testing period and a higher testing cost, making it unsuitable as the first detection method for every cancer type.

### DNA‐based NGS

Nowadays, in clinical practice, DNA‐based NGS is a widely applied cancer genetic test. In a clinical study, the specificity of a DNA‐based NGS test was 99.86%, with an overall sensitivity of 81.1% (the sensitivity for *NTRK1* gene fusions was 96.8%, but was relatively lower for *NTRK2/3* gene fusions).^22^ Other than whole genome sequencing, panel and whole exome sequencing (WES) are both based on target region enrichment, including the amplicon‐based and hybrid capture‐based methods. For amplicon‐based methods, the exact sequence of partner genes is required, making it unsuitable for *NTRK* gene fusion detection, where numerous partner genes and complex fusion patterns exist. In contrast, hybrid capture‐based methods do not require known partner genes. Simply designing probes for *NTRK* gene kinase domain regions, where breakpoints are commonly found, can help researchers achieve the detection of known and unknown *NTRK* gene fusions. However, due to the extremely large breakpoint region within the introns of *NTRK2* and (especially) *NTRK3*, which consist of a large number of repeating regions that are not suitable for the design of hybrid capture probes, in practice, partner genes, such as *ETV6* and *EML4*, are used for probe design. The low coverage of probes may lead to false‐negatives.^40^ Notably, panels in the market place have different coverage for *NTRK* genes. As such, special attention is required when they are used.

### RNA‐based NGS

RNA‐based NGS is the best method for detecting *NTRK* gene fusions. Since at RNA level NGS directly detects transcripts following splicing, RNA‐based NGS can serve as a method for reconfirming whether or not nonclassic *NTRK* structural variants detected by DNA‐based NGS generated oncogenic fusion RNA. Also, RNA‐based NGS can help researchers avoid the technical problem of DNA‐based NGS caused by fusions within intron regions. RNA‐based NGS is generally divided into three types: (1) whole transcriptome sequencing (WTS), (2) hybrid capture, and (3) anchored multiplex polymerase chain reaction (AMP). Of these three types of RNA‐based NGS, WTS and hybrid capture can determine gene expression level in additional to fusion genes. AMP enriches target regions via a single direction *NTRK*‐specific adapter and a universal adapter added to complementary DNA, making it possible to detect unknown fusions with a high degree of sensitivity.^41^, ^42^, ^43^ Unfortunately, RNAs are highly unstable and easily degraded. As such, the detection of RNA in formalin‐fixed paraffin‐embedded (FFPE) tissues is challenging.^35^ Indeed, a study that performed RNA‐based NGS on 44 FFPE specimens revealed that only 52.3% (23/44) of specimens passed a presequencing quality control assessment.^44^

Pros and cons exist for DNA‐ and RNA‐based NGS tests. Simultaneously performing both DNA and RNA tests can enhance the detection rate and the accuracy of *NTRK* gene fusions. To mutually integrate the two methods, several protocols are currently undergoing clinical validation.^45^, ^46^, ^47^ Moreover, when tumor tissue specimens cannot be obtained, plasma circulating tumor DNA (ctDNA)‐based NGS test can serve as an accurate and clinically applicable surrogate method.^48^ Due to the high number of partner genes, the *NTRK* gene fusion probe region requires high coverage. However, ctDNA panels normally target limited probe regions to ensure a high sequencing depth, which may result in an increase in false‐negatives. Indeed, a study that applied DNA‐based NGS to ctDNA samples collected from lung cancer patients indicated that the sensitivity of the *ALK* gene fusion was merely 54.2%.^49^ Considering the relative high sensitivity of ctDNA tests for detecting single nucleotide variants (SNVs), detecting drug resistant mutations during a TRK inhibitor treatment course in patients with *NTRK* gene fusions is recommended as a surveillance method.

## THE CLINICAL APPLICATION OF *NTRK* GENE FUSIONS

TRK inhibitors are the first agnostic tumor targeted therapy approved by the regulatory authority, and are the best model for tumor precision medicine, especially for the development of a novel “basket trial.”^50^ Larotrectinib and entrectinib are the first FDA‐approved TRK inhibitors for treating pan‐cancer patients with *NTRK1/2/3* gene fusions. Establishing standards and consensus for *NTRK1/2/3* gene fusion diagnosis and treatment in various cancer types is, therefore, key for applying TRK inhibitors in actual clinical practice.

### Larotrectinib

Given the efficacy and tolerability of larotrectinib, it has been approved by the FDA and the EMA for treating *NTRK* gene fusion‐positive adult and pediatric patients with solid tumors that are locally progressed or metastatic, unresectable, or that have no satisfactory alternative treatment available. Different dosage forms, including capsules and oral solutions, were approved by NMPA in April and June 2022, respectively, for treating *NTRK* gene fusion‐positive adult and pediatric patients with locally advanced or metastatic solid tumors. A pan‐cancer clinical trial demonstrated that the objective response rate (ORR) for larotrectinib was 75% in a population of 55 cancer patients with 17 different types of *NTRK* gene fusions and an age ranging from 4 months to 76 years.^31^ Updated data was reported in ASCO 2022. In a population of 244 *NTRK* gene fusion‐positive cancer patients spanning 25 cancer types, larotrectinib displayed an ORR of 69% (95% CI: 62–79) and the complete response (CR) rate was 26%. When the median follow‐up duration was 28.3 months, the median duration of response (DoR) was 32.9 months (95% CI: 27.3–41.7). When the median follow‐up duration was 29.3 months, the median progression‐free survival (PFS) was 29.4 months (95% CI: 19.3–34.3), the 48‐month overall survival (OS) rate was 64% (95% CI: 55–73), and adverse effects were mainly grade 1–2, suggesting good tolerability.^51^ Larotrectinib also displayed efficacy in both first‐line and late‐line settings, with an ORR of 91% and ≥70%, respectively.^52^ For *NTRK* gene fusion‐positive cancer patients under 18 years (noncentral nervous system [CNS] cancer), a study where most of the patients had sarcoma revealed that the ORR of larotrectinib reached 84% (95% CI: 75–91).^53^ For *NTRK* gene fusion‐positive lung cancer patients, the ORR was 83% (95% CI: 61–95).^54^

### Entrectinib

Entrectinib has been approved by the FDA for treating patients 12 years of age and older with *NTRK* gene fusion‐positive solid tumors that are metastatic, unresectable, or that have no satisfactory alternative treatment available. In July 2022, the NMPA subsequently approved entrectinib for treating adult and pediatric patients 12 years of age and older with *NTRK* gene fusion‐positive, locally advanced or metastatic solid tumors. Entrectinib is also a multitarget drug that can be used to treat *ROS1* and *ALK* gene fusion‐positive cancer patients. Recently, in ASCO 2022, a study reported updated clinical data for entrectinib in 150 adult patients with *NTRK* gene fusion‐positive, locally advanced, or metastatic solid tumors covering 17 cancer types (only patients with a follow‐up period of ≥12 months were included in the analysis). In the study, the ORR was 61.3% (95% CI: 53.1–69.2) and the CR rate was 16.7% (*n* = 25). When the median follow‐up duration was 30.6 months, the median DoR was 20.0 (95% CI: 13.2–31.1), the median PFS was 13.8 months (95% CI: 10.1–20.0), the median OS was 37.1 months (95% CI: 27.2‐not estimable), and adverse effects were mainly grade 1–2. Interestingly, the ORR for entrectinib in patients with (61.3%; 95% CI: 42.2–78.2) and without (61.3%; 95% CI: 52.0–70.1) baseline CNS metastases was comparable.^55^

### Drug resistance and second‐generation TRK inhibitors

Similar to other targeted drugs, patients may develop a resistance to TRK inhibitors during the course of treatment. Known variants that cause a resistance to first‐generation TRK inhibitors include: (1) solvent‐front mutations, such as *NTRK1* G595R and *NTRK3* G623R; (2) gatekeeper mutations, such as *NTRK1* F589L; (3) xDFG motif mutations, such as *NTRK1* G667C and *NTRK3* G696A; (4) bypass pathway mutations, such as *BRAF* mutations, IGF1R activation, *KRAS* mutations, and MET amplifications; and (5) other or unknown mutations, such as *NTRK1* A608D. Among variants, solvent‐front mutations are the most commonly observed resistance mechanism, comprising approximately 45% of patients with progression following treatment with TRK inhibitors or patients that are TRK inhibitor intolerant.^12^, ^56^ To precisely screen for responding patients and to guide treatment decisions following progression, performing genetic tests for known resistance‐related variants prior to treatment and following the progression of first‐generation TRK inhibitors is recommended.

To overcome drug resistance to first‐generation TRK inhibitors, second‐generation TRK inhibitors are under development and are currently a part of clinical trials. Selitrectinib is specifically designed to overcome first‐generation drug resistance caused by mutations of the TRK kinase domain, and is currently in a phase I/II clinical trial. Repotrectinib is a multikinase inhibitor that can inhibit the activity of *NTRK*, *ROS1*, and *ALK* gene fusions. Repotrectinib is currently undergoing a phase I/II clinical trial to study its effects in treating patients with *NTRK* gene fusion‐positive, tyrosine kinase inhibitor‐pretreated advanced solid tumors. The FDA has granted repotrectinib a breakthrough therapy designation. Preclinical data has demonstrated satisfying results for both selitrectinib and repotrectinib to solvent‐front and gatekeeper mutations.^57^, ^58^, ^59^ Preliminary data from clinical trials has consistently suggested the efficacy of selitrectinib and repotrectinib in treating patients who have progressed following first‐generation TRK inhibitors. The ORR for selitrectinib in the entire population and in TRK kinase mutated (on‐target) patients was 34% (10/29) and 45% (9/20), respectively.^55^ For repotrectinib, the ORR was 50% (3/6) for the entire population.^60^ Other drugs targeting *NTRK1/2/3* gene fusions are currently under development. Their major clinical trials are provided in Supporting Information Table S2. Additionally, NTRK gene fusions have been reported as acquired resistance in patients who had undergone multi‐line therapies^61^, indicating that TRK inhibitors may be effective in such clinical setting.

## DISCUSSION

**FIGURE 2:**
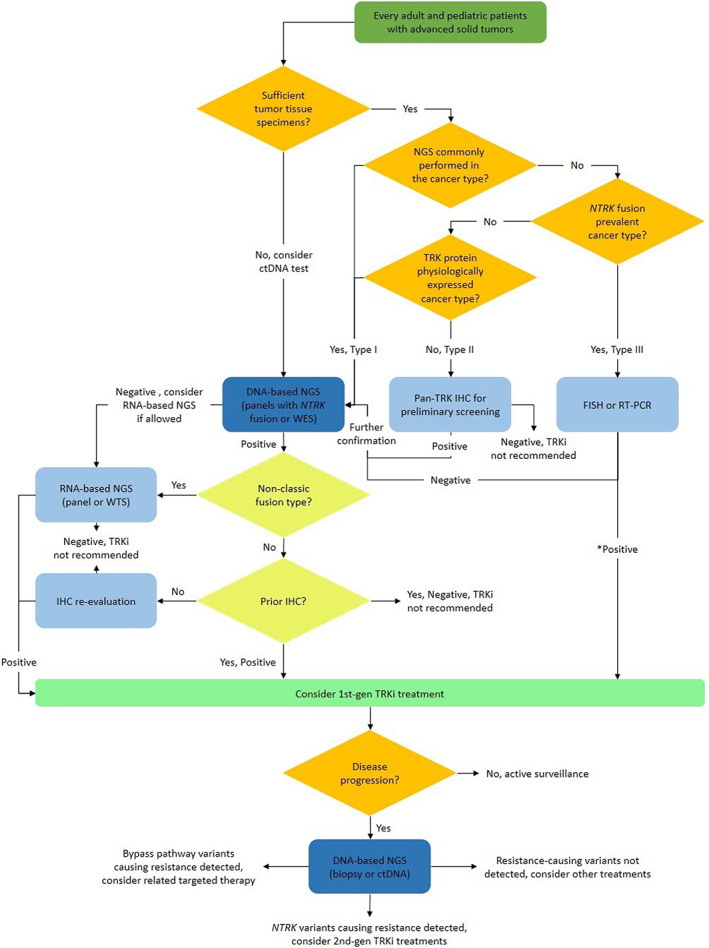
The recommended procedure for the diagnosis and treatment of *NTRK* gene fusion solid tumors. *For positive FISH results, reconfirmation by NGS as to whether or not the detected structural variant events have a biological function is recommended. FISH, fluorescence in situ hybridization; IHC, immunohistochemistry; NGS, next‐generation sequencing; RT‐PCR, reverse transcription‐polymerase chain reaction; TRK, tyrosine receptor kinase; WES, whole exome sequencing; WTS, whole transcriptome sequencing. To clearly show the flow of different parts.

Our expert group discussed the above‐mentioned issues and established 13 consensus on the diagnosis and treatment of *NTRK* gene fusion solid tumors in China (Table 2). We also determined a recommended procedure for the diagnosis and treatment of *NTRK* gene fusion solid tumors (Figure 2). To recommend suitable *NTRK* gene fusion tests and to provide treatment suggestions based on the needs of particular patients, our group additionally classified cancer into three types: (1) whether or not NGS is commonly performed, (2) whether or not the TRK protein is physiologically expressed, and (3) whether or not *NTRK* gene fusions are prevalent (Table 3).

**TABLE 2:** Consensus on the diagnosis and treatment of *NTRK* gene fusion solid tumors in China

	Consensus number	Key points	Recommendation level
Detection time point	Consensus 1	A *NTRK* gene fusion test is recommended for every advanced adult and pediatric solid tumor patient. Different cancer types should be tested using a specific detection strategy	Strongly recommended
	Consensus 2	Advanced adult and pediatric solid tumor patients should consider a *NTRK* gene fusion test prior to the initiation of standard treatment or during treatment. For locally advanced cancer patients with *NTRK* gene fusion prevalent cancer types, a *NTRK* gene fusion test should be performed before neoadjuvant treatment	Strongly recommended
Detection method	Consensus 3	A DNA‐based NGS panel with the *NTRK*'s intron region covered or a WES is recommended as the main method for *NTRK* gene fusion detection. An RNA‐based NGS panel or a WTS serves as an important supplementary method for *NTRK* gene fusion detection. An RNA‐based NGS panel possesses higher sensitivity than a DNA‐based NGS panel. Especially when IHC‐positive and, DNA‐based NGS panel‐negative, an RNA‐based NGS panel is recommended for reconfirmation. When possible, simultaneously extracting DNA and RNA from FFPE slices, for the purpose of performing concurrent DNA‐ and RNA‐based NGS tests, is recommended	Strongly recommended
	Consensus 4	*ETV6‐NTRK3* FISH can serve as a confirmation test in cancer types with a high mutational frequency of *NTRK* gene fusions, but is not recommended for other cancer types. For positive cases, reconfirmation by NGS as to whether or not the detected structural variant events have a biological function is recommended	Recommended
	Consensus 5	RT‐PCR can serve as a confirmation test in cancer types with a high mutational frequency of *NTRK* gene fusions and known partner genes (for example, an *ETV6‐NTRK3* fusion), but is not recommended for other cancer types. Using NGS as a reconfirmation method in patients with negative results for RT‐PCR is recommended	Recommended
	Consensus 6	Pan‐TRK IHC can serve as a screening method in cancer types with a low mutational frequency for *NTRK* gene fusion, not expressing *NTRK*, when a DNA‐based NGS panel test is not commonly performed. Also, pan‐TRK IHC can serve as a re‐evaluation method for NGS tests	Recommended
Detection strategy	Consensus 7	According to the prevalence of *NTRK* gene fusions, physiological expression of the TRK protein, and the popularity of NGS tests (30% as cut‐off threshold), classifying cancers into three types and performing a *NTRK* gene fusion test, as stated in Figure 2, is recommended	Recommended
	Consensus 8	Establishing a standard procedure for detecting *NTRK* in individual hospitals is recommended. Our expert group will routinely release recommendations regarding the significance of NGS testing for each cancer type. In light of the fast development of cancer precision medicine, with *NTRK* gene fusion detection as a marker, actively promoting the development of precision medicine in hospitals at different levels is important	Recommended
Detection quality control	Consensus 9	All tests should be performed in medical‐certified laboratories. Choosing authority‐certified laboratories such as those that are ISO15189‐, CAP‐, CLIA‐certified to perform tests, is recommended. Laboratories should launch *NTRK* external and internal quality controls according to related regulations	Strongly recommended
	Consensus 10	Only FFPE tumor specimens that passed pathological quality control can be used to perform *NTRK* gene fusion tests. When tumor tissue cannot be obtained or when an insufficient tumor cell content is present in FFPE specimens, peripheral blood can be considered for ctDNA test in advanced cancer patients. For RNA tests, fresh tissues are generally the best material. If sufficient fresh tissues with a satisfactory tumor tissue/cell content can be obtained, DNA + RNA NGS tests can be considered. Lack of sampling experience could increase the risk of insufficient tumor cell content. We recommend bathing biopsy tissues in 10% formalin for 6–12 h and bathing surgical tissues for 24–48 h. When FFPE specimens are used to perform DNA + RNA NGS tests, recently obtained specimens are recommended (using specimen obtained over 2 years old is not recommended). Selected splices should contain a sufficient amount of tumor cells to meet the requirement for different detection platforms. When performing IHC or FISH, at least 50 tumor cells are required. When performing RT‐PCR, at least 5% tumor cells are required. When performing NGS, the tumor cell content of the sample should be at least 20% If the tumor cell content of splices are not sufficient, microscopic dissection can be considered to enrich tumor tissues. When cutting tumor specimens from different patients, the blade and the spreading pool water should be cleaned or replaced to avoid cross‐contamination DNA‐based NGS tests for *NTRK* gene fusions should clearly state the probe region covered (including introns). To avoid false‐negatives, choosing tests with intact probe coverage or simultaneous RNA‐based NGS tests are recommended	Strongly recommended
	Consensus 11	In addition to basic information and quality control data, test reports should include indicators such as tumor cell content, microscopic dissection status, and the concentration and purity of extracted DNA. For NGS test reports, positive fusion results should include data such as the breakpoint position in the chromosome, the involvement of tyrosine kinase domain, and in‐frame fusion. When the *NTRK* gene fusion involves tyrosine kinase domain and is in‐frame fusion, it should be reported as fusion, otherwise, reported as rearrangement	Strongly recommended
	Consensus 12	When a physician is in doubt (for example, inconsistent results in different tests, novel partner genes or fusion patterns, complex fusion events, in‐frame fusion or involvement of intact tyrosine kinase domain cannot be confirmed, multiple driver genes positive, etc.), discussing results and future treatment decisions with the Molecular Tumor Board (MTB) is recommended	Strongly recommended
Treatment strategy	Consensus 13	For *NTRK* gene fusion‐positive patients with solid tumors, treatment with TRK inhibitors such as Larotrectinib and Entrectinib, or participation in TRK inhibitor‐related clinical trials is recommended. For *NTRK* gene fusion‐positive tumor patients with drug resistance, performing NGS tests to identify resistant‐causing mutations and deciding whether or not second‐generation TRK inhibitors or related clinical trials are appropriate are recommended	Strongly recommended

Abbreviations: FFPE, formalin‐fixed paraffin‐embedded; FISH, fluorescence in situ hybridization; IHC, immunohistochemistry; NGS, next‐generation sequencing; RT‐PCR, reverse transcription‐polymerase chain reaction; TRK, tyrosine receptor kinase; WTS, whole transcriptome sequencing.

**TABLE 3:** The classification of cancer types for guiding *NTRK* gene fusions detection strategy

Classification	NGS popularity	TRK protein physiological expression	Fusion prevalence	Representative cancer types	Recommended detection method
Type I	High	−	Low	Non‐small cell lung cancer, triple‐negative breast cancer, colorectal cancer, prostate cancer, cholangiocarcinoma, melanoma, ovarian cancer	A DNA‐based NGS panel with *NTRK* covered or a WES is recommended; a combination of RNA‐based NGS tests is recommended; nonclassic fusion pattern should be further validated via pan‐TRK IHC
	Low	+	Low	Brain tumors such as glioma, gastrointestinal stromal tumor, soft tissue sarcoma, neuroendocrine tumor	
Type II	Low	−	Low	Nontriple‐negative breast cancer, liver cancer, gastric cancer, cervical cancer	Pan‐TRK IHC is first applied for screening; positive cases should be reconfirmed via DNA/RNA NGS
Type III	Low	−	High	Infantile fibrosarcoma, secretory salivary gland carcinoma, secretory breast cancer, congenital mesoblastic nephroma	FISH or RT‐PCR are recommended for confirmation; negative cases should be reconfirmed via NGS testing

Abbreviations: FISH, fluorescence in situ hybridization; IHC, immunohistochemistry; NGS, next‐generation sequencing; RT‐PCR, reverse transcription‐polymerase chain reaction; TRK, tyrosine receptor kinase; WES, whole exome sequencing.

As the first targeted therapy‐related biomarker approved by the regulatory authority for pan‐cancer use, the detection strategy for *NTRK* gene fusions is key in cancer precision medicine. Despite issues in health economics and real‐world practice, the most suitable detection method for *NTRK* gene fusion is DNA + RNA NGS test. In actual clinical practice, such a detection strategy is associated with the popularity of NGS, the value of multigene testing in each cancer type, patient economic condition, the knowledge of molecular pathological testing at each hospital, and, also, *NTRK* gene fusion prevalence and the possibility of screening with pan‐TRK IHC (TRK protein physiological expression).

*NTRK* gene fusion detection is complicated and can be key in the clinical practice of cancer precision medicine. The writing, spread, and popularization of consensus have important clinical significance. However, the recommended detection strategy provided in this consensus is unavoidably impacted by the regulatory policy of the related health inspection field and requires experts in this area to actively apply this expert consensus, industry standards, and guidelines in order to impact the launch of beneficial policies. Due to the lack of released and related research in China, most of the research cited in this consensus was obtained from foreign countries. To a certain extent, this lack of information limits evidence‐based strength for applying this consensus in China. Also, part of the content in this consensus, especially contents related to quality control, lacks large‐scale clinical trials as supporting evidence. Therefore, related consensus information requires further validation through future studies.

## CONFLICT OF INTEREST

There are no conflicts of interest to disclose.

## Supporting information


**Table S1** List of known partner genes

**Table S2** *NTRK1/2/3* inhibitors under development
