# Supplementary material for: Expert consensus on the diagnosis and treatment of NTRK gene fusion solid tumors in China
Source: Thorac Cancer. 2022 Sep 20;13(21):3084–97. doi: 10.1111/1759-7714.14644 (PMC9626341; doi:10.1111/1759-7714.14644)
Supplement: Supplementary file 1 — Table S1 List of known partner genes [file TCA-13-3084-s002.docx]

Supplementary table 1. List of known partner genes

| *NTRK* gene (chromosomal location) | Classic partner genes | Chromosomal location of classic partner genes | Rare partner genes | Chromosomal location of rare partner genes |
| --- | --- | --- | --- | --- |
| *NTRK1* (1q23.1) | *MPRIP* | 17p11.2 | *ACO1* | 9p21.1 |
|  | *TP53* | 17p13.1 | *DYNC2H1* | 11q22.3 |
|  | *TFG* | 3q12.2 | *MIR548F1* | 10q21.1 |
|  | *TPR* | 1q31.1 | *EGFR* | 7p11.2 |
|  | *IRF2BP2* | 1q42.3 | *EML4* | 2p21 |
|  | *LMNA* | 1q22 | *AGFG1* | 2q36.3 |
|  | *NFASC* | 1q32.1 | *CCDC88C* | 14q32.11 |
|  | *TPM3* | 1q21.3 | *EPHB2* | 1p36.12 |
|  | *SFPQ* | 1p34.3 | *ARGLU1* | 13q33.3 |
|  | *EPS15* | 1p32.3 | *ARHGEF11* | 1q23.1 |
|  | *AFAP1* | 4p16.1 | *ARHGEF25* | 12q13.3 |
|  | *ARHGEF2* | 1q22 | *ATP1B1* | 1q24.2 |
|  | *BCAN* | 1q23.1 | *BGLAP* | 1q22 |
|  | *CD74* | 5q32 | *GON4L* | 1q22 |
|  | *CHTOP* | 1q21.3 | *GRIPAP1* | Xp11.23 |
|  | *CTRC* | 1p36.21 | *CABLES1* | 18q11.2 |
|  | *PPL* | 16p13.3 | *CD5* | 11q12.2 |
|  | *SCYL3* | 1q24.2 | *CDC42BPA* | 1q42.13 |
|  | *SQSTM1* | 5q35.3 | *PIP5K1A* | 1q21.3 |
|  | *TRIM63* | 1p36.11 | *CEL* | 9q34.2 |
|  |  |  | *CGN* | 1q21.3 |
|  |  |  | *KCTD16* | 5q31.3 |
|  |  |  | *KIF21B* | 1q32.1 |
|  |  |  | *CPSF7* | 11q12.2 |
|  |  |  | *PRDX1* | 1p34.1 |
|  |  |  | *RNF213* | 17q25.3 |
|  |  |  | *CUL4A* | 13q34 |
|  |  |  | *PTPRC* | 1q31.3 |
|  |  |  | *DCST1* | 1q22 |
|  |  |  | *DCTN1* | 2p13.1 |
|  |  |  | *RABGAP1L* | 1q25.1 |
|  |  |  | *MDM4* | 1q32.1 |
|  |  |  | *DUSP10* | 1q41 |
|  |  |  | *MEX3A* | 1q22 |
|  |  |  | *TRIM33* | 1p13.2 |
|  |  |  | *EFNA3* | 1q22 |
|  |  |  | *MRPL24* | 1q23.1 |
|  |  |  | *MTA1* | 14q32.33 |
|  |  |  | *NRG1* | 8p12 |
|  |  |  | *PDE4DIP* | 1q21.1 |
|  |  |  | *RPL7A* | 9q34.2 |
|  |  |  | *ERC1* | 12p13.33 |
|  |  |  | *SCP2* | 1p32.3 |
|  |  |  | *NECTIN4* | 1q23.3 |
|  |  |  | *GATAD2B* | 1q21.3 |
|  |  |  | *AMOTL2* | 3q22.2 |
|  |  |  | *NOS1AP* | 1q23.3 |
|  |  |  | *PHF20* | 20q11.22-q11.23 |
|  |  |  | *SMG5* | 1q22 |
|  |  |  | *LIPI* | 21q11.2 |
|  |  |  | *CACYBP* | 1q25.1 |
|  |  |  | *PEAR1* | 1q23.1 |
|  |  |  | *RAB25* | 1q22 |
|  |  |  | *CACNA1E* | 1q25.3 |
|  |  |  | *PLEKHA6* | 1q32.1 |
|  |  |  | *KHDRBS1* | 1p35.1 |
|  |  |  | *GP2* | 16p12.3 |
|  |  |  | *KIRREL1* | 1q23.1 |
|  |  |  | *LRRC71* | 1q23.1 |
|  |  |  | *ZBTB7B* | 1q21.3 |
|  |  |  | *SEL1L* | 14q31.1 |
|  |  |  | *NLGN1* | 3q26.31 |
|  |  |  | *RCSD1* | 1q24.2 |
|  |  |  | *NAB2* | 12q13.3 |
|  |  |  | *SEMA4B* | 15q26.1 |
|  |  |  | *PRKAR1A* | 17q24.2 |
|  |  |  | *UBAP2L* | 1q21.3 |
|  |  |  | *SPTA1* | 1q23.1 |
|  |  |  | *IGSF8* | 1q23.2 |
|  |  |  | *STRN3* | 14q12 |
|  |  |  | *TPM1* | 15q22.2 |
|  |  |  | *ZBTB1* | 14q23.3 |
|  |  |  | *SSBP2* | 5q14.1 |
|  |  |  | *ATP8B2* | 1q21.3 |
| *NTRK2* (9q21.33) | *AFAP1* | 4p16.1 | *HOOK3* | 8p11.21 |
|  | *SQSTM1* | 5q35.3 | *EML4* | 2p21 |
|  | *AGBL4* | 1p33 | *MTMR2* | 11q21 |
|  | *BCR* | 22q11.23 | *SPECC1L* | 22q11.23 |
|  | *PAN3* | 13q12.2 | *DAB2IP* | 9q33.2 |
|  | *QKI* | 6q26 | *PCSK5* | 9q21.13 |
|  | *TRAF2* | 9q34.3 | *CTDSP2* | 12q14.1 |
|  | *TRIM24* | 7q33-34 | *LAP3* | 4p15.32 |
|  | *VCL* | 10q22.2 | *HMBOX1* | 8p21.1-p12 |
|  |  |  | *GNAQ* | 9q21.2 |
|  |  |  | *ACO1* | 9p21.1 |
|  |  |  | *DENND1A* | 9q33.3 |
|  |  |  | *PAIP1* | 5p12 |
|  |  |  | *TRIP13* | 5p15.33 |
|  |  |  | *NAV1* | 1q32.1 |
|  |  |  | *SLC28A3* | 9q21.32-q21.33 |
|  |  |  | *TP63* | 3q28 |
|  |  |  | *RASEF* | 9q21.32 |
|  |  |  | *GKAP1* | 9q21.32 |
|  |  |  | *FAM117B* | 2q33.2 |
|  |  |  | *THADA* | 2p21 |
|  |  |  | *NOD1* | 7p14.3 |
|  |  |  | *SLMAP* | 3p14.3 |
|  |  |  | *RBPMS* | 8p12 |
|  |  |  | *NACC2* | 9q34.3 |
|  |  |  | *PRRX1* | 1q24.2 |
|  |  |  | *PPP6R3* | 11q13.2 |
|  |  |  | *PML* | 15q24.1 |
|  |  |  | *STRN* | 2p22.2 |
|  |  |  | *UFD1* | 22q11.21 |
| *NTRK3* (15q25.3) | *ETV6* | 12p13.2 | *AMMECR1* | Xq23 |
|  | *BTBD1* | 15q25.2 | *CDK12* | 17q12 |
|  | *KHDRBS1* | 1p35.1 | *HOMER1* | 5q14.1 |
|  | *MYO5A* | 15q21.2 | *MEF2A* | 15q26.3 |
|  | *PLEKHA6* | 1q32.1 | *RORA* | 15q22.2 |
|  | *SQSTM1* | 5q35.3 | *KANK1* | 9p24.3 |
|  | *TPM4* | 19p13.12 | *PEAK1* | 15q24.3 |
|  |  |  | *IQGAP1* | 15q26.1 |
|  |  |  | *SPECC1L* | 22q11.23 |
|  |  |  | *DLG1* | 3q29 |
|  |  |  | *FAT1* | 4q35.2 |
|  |  |  | *CHST11* | 12q23.3 |
|  |  |  | *LYN* | 8q12.1 |
|  |  |  | *COX5A* | 15q24.2 |
|  |  |  | *AKAP13* | 15q25.3 |
|  |  |  | *EML4* | 2p21 |
|  |  |  | *MYH9* | 22q12.3 |
|  |  |  | *TNRC6A* | 16p12.1 |
|  |  |  | *PDE8A* | 15q25.3 |
|  |  |  | *LOXL2* | 8p21.3 |
|  |  |  | *RALGPS2* | 1q25.2 |
|  |  |  | *PML* | 15q24.1 |
|  |  |  | *SLC8B1* | 12q24.13 |
|  |  |  | *TFG* | 3q12.2 |
|  |  |  | *AGBL1* | 15q25.3 |
|  |  |  | *HMBOX1* | 8p21.1-p12 |
|  |  |  | *VPS18* | 15q15.1 |
|  |  |  | *ZBTB10* | 8q21.13 |
|  |  |  | *RUNX1* | 21q22.12 |
|  |  |  | *ZSCAN2* | 15q25.2 |
|  |  |  | *RAD52* | 12p13.33 |
|  |  |  | *LINC00924* | 15q26.2 |
|  |  |  | *EFL1* | 15q25.2 |
|  |  |  | *STRN* | 2p22.2 |
|  |  |  | *FANK1* | 10q26.2 |
|  |  |  | *ABHD2* | 15q26.1 |
|  |  |  | *LRRC28* | 15q26.3 |
|  |  |  | *SPTAN1* | 9q34.11 |
|  |  |  | *HERC1* | 15q22.31 |
|  |  |  | *SCAPER* | 15q24.3 |
|  |  |  | *STK3* | 8q22.2 |
|  |  |  | *PKM* | 15q23 |
|  |  |  | *STRN3* | 14q12 |
|  |  |  | *SPTBN1* | 2p16.2 |
|  |  |  | *RBPMS* | 8p12 |
|  |  |  | *NBEA* | 13q13.3 |
|  |  |  | *MCTP2* | 15q26.2 |
|  |  |  | *SH3GL3* | 15q25.2 |
|  |  |  | *COL8A1* | 3q12.1 |
|  |  |  | *MORF4L1* | 15q25.1 |
|  |  |  | *PPFIA2* | 12q21.31 |
|  |  |  | *PTEN* | 10q23.31 |
|  |  |  | *THSD4* | 15q23 |
|  |  |  | *LRRK1* | 15q26.3 |

*Classic and rare partner genes are defined based on <http://quiver.archerdx.com/>.
