# Supplementary material for: Expert consensus on the diagnosis and treatment of NTRK gene fusion solid tumors in China
Source: Thorac Cancer. 2022 Sep 20;13(21):3084–97. doi: 10.1111/1759-7714.14644 (PMC9626341; doi:10.1111/1759-7714.14644)
Supplement: Supplementary file 2 — Table S2 NTRK1/2/3 inhibitors under development [file TCA-13-3084-s001.docx]

**Supplementary table 2: *NTRK1/2/3* inhibitors under development**

| Drug ID | Generic name | Generation | Target(s) | Phase | Clinical Trial ID |
| --- | --- | --- | --- | --- | --- |
| LOXO-101 | Larotectinib | First | TRK | FDA/  NMPA approved | NA |
| RXDX-101 | Entrectinib | First | TRK/ROS1/ALK | FDA/  NMPA approved | NA |
| FCN011 | NA | First | TRK | I/II | NCT04687423 |
| TL118 | NA | First | TRK | I | CTR20191622 |
| TQB-3558 | NA | First | TRK | I | NCT04408079 |
| PLX7486 | NA | First | TRK/CSF1R | I | NCT01804530(terminated) |
| LOXO-195 (BAY2731954) | Selitrectinib | Second | TRK | I/II | NCT03215511 |
| TPX-005 | Repotrectinib | Second | TRK/ROS1/ALK | I/II | NCT04094610 NCT03093116 |
| PBI-200 | NA | Second | TRK | I/II | NCT04901806 |
| VC004 | NA | Second | TRK | I/II | NCT04614740 (CTR20201703) |
| XZP-5955 | NA | Second | TRK/ROS1 | I/II | NCT04996121 (CTR20211858) |
| DS-6051b (AB-106 in China) | Taletrectinib | Second | TRK/ROS1 | I | NCT02279433(completed) (CTR20202099-recruiting) |
| FCN098 | NA | Second | TRK | I | NCT05212987 |
| SIM-1803 | NA | Second | TRK/ROS1/ALK | I | NCT04671849 |
| VMD-928 | NA | Second | TRKA | I | NCT03556228 |
| ICP-723 | NA | Second | TRK | I/II | NCT04685226 (CTR20202270) |
| TSR-011 | Belizatinib | Second (ALK) | TRK/ALK | I/II | NCT02048488(completed) |
| BPI-28592 | NA | Second | TRK | I | NCT05302843 (CTR20202486) |
| HG030 | NA | Second | TRK/ROS1 | I | CTR20202020 |
| TQB-3811 | NA | Second | TRK | I | NCT05046847 |
| ONO-7579 | NA | Second | TRK | I | NCT03182257(terminated) |
| LPM4870108 | NA | Second | TRK | CDE-IND | ？ |
| ONO-5390556 | NA | Second | TRK | ？ | ？ |
| LY2801653 | Merestinib | NA | MST1R/FLT3/AXL/MERTK/TEK/ROS1/TRK/DDR/MKNK | II | NCT02920996 |
| MGCD516 | Sitravatinib | NA | MET/AXL/MER/VEGFR/PDGFR/DDR2/TRK/Eph | I | NCT02219711 |
| TSN084 | NA | NA | MET/FLT3/TRK/CDK8/CDK19 | I | NCT05300438 (CTR20220834) |
| DCC-2701 | Altiratinib | NA | MET/TIE2/VEGFR2/FLT3/TRK | I | NCT02228811(terminated) |
| CEP-701 | Lestaurtinib | NA | JAK2/FLT3/TRKA | ？ | ？ |
